# The roles of experienced and internalized weight stigma in healthcare experiences: Perspectives of adults engaged in weight management across six countries

**DOI:** 10.1371/journal.pone.0251566

**Published:** 2021-06-01

**Authors:** Rebecca M. Puhl, Leah M. Lessard, Mary S. Himmelstein, Gary D. Foster

**Affiliations:** 1 Department of Human Development & Family Sciences, University of Connecticut, Storrs, Connecticut, United States of America; 2 Rudd Center for Food Policy and Obesity, University of Connecticut, Hartford, Connecticut, United States of America; 3 Department of Psychological Sciences, Kent State University, Kent, Ohio, United States of America; 4 WW, New York, New York, United States of America; 5 Center for Weight and Eating Disorders, Perelman School of Medicine, University of Pennsylvania, Philadelphia, Pennsylvania, United States of America; University College London, UNITED KINGDOM

## Abstract

**Background/Objectives:**

Considerable evidence from U.S. studies suggests that weight stigma is consequential for patient-provider interactions and healthcare for people with high body weight. Despite international calls for efforts to reduce weight stigma in the medical community, cross-country research is lacking in this field. This study provides the first multinational investigation of associations between weight stigma and healthcare experiences across six Western countries.

**Methods:**

Participants were 13,996 adults residing in Australia, Canada, France, Germany, the UK, and the US who were actively enrolled in an internationally available behavioral weight management program. Participants completed identical online surveys in the dominant language for their country that assessed experienced weight stigma, internalized weight bias, and healthcare behaviors and experiences including perceived quality of care, avoidance or delay of seeking care, experiences with providers, and perceived weight stigma from doctors.

**Results:**

Among participants who reported a history of weight stigma (56–61%), two-thirds of participants in each country reported experiencing weight stigma from doctors. Across all six countries, after accounting for demographics, BMI, and experienced stigma, participants with higher internalized weight bias reported greater healthcare avoidance, increased perceived judgment from doctors due to body weight, lower frequency of obtaining routine checkups, less frequent listening and respect from providers, and lower quality of healthcare. Additionally, experienced weight stigma (from any source) was indirectly associated with poorer healthcare experiences through weight bias internalization, consistently across the six countries.

**Conclusions:**

Weight stigma in healthcare is prevalent among adults actively engaged in weight management across different Western countries, and internalized weight bias has negative implications for healthcare even after controlling for BMI. The similar findings across all six countries underscore the negative consequences of weight stigma on healthcare behaviors and experiences, and emphasize the need for collective international efforts to address this problem.

## Introduction

High rates of obesity around the world [1] have garnered sustained attention and efforts from medical, public health, and scientific communities. Simultaneously, scholars across diverse social science disciplines have studied the pervasive societal stigma faced by individuals with higher body weight. Known as weight stigma, individuals with higher weight face numerous negative stereotypes, prejudice, and unfair treatment across multiple facets of everyday life including healthcare [2]. Despite increasing obesity rates in recent decades, research suggests that weight bias and stigma have worsened, rather than improved over time [3], even among obesity specialists [4]. Furthermore, evidence documenting the presence of weight stigma in many countries [5–7], and its harmful health consequences [8,9], has led to increasing recognition that weight stigma itself is a global health issue [10].

The health harms of weight stigma include numerous negative consequences for psychological wellbeing [8,9] and physical health [11–13], all of which accentuate the need for appropriate healthcare and treatment to both adequately support individuals who face weight stigma and diminish the adverse health effects incurred from this stigma. However, research spanning several decades has documented the presence of weight stigma in the healthcare setting [13–15], creating additional challenges and barriers to quality care for patients with higher weight. To date, most of this research examining associations between weight stigma and healthcare experiences of individuals with higher weight has come from the US. This evidence has identified a myriad of ways that weight stigma permeates the healthcare environment. In particular, studies have consistently documented negative weight-based stereotypes and attitudes reported by healthcare providers [16,17], including evidence that doctors express both implicit and explicit weight bias at levels similar to the general population [18]. These findings parallel reports from patients with higher body weight, who identify doctors as one of the most common interpersonal sources of weight stigma [19,20].

Additionally, weight stigma has concerning implications for patient-provider interactions and patients’ healthcare utilization, as evidenced in US studies [10,15,21]. People who feel judged about their weight from a doctor report lower quality interactions with healthcare providers [22], less frequent clinician-patient interactions [22], lower trust in their primary care provider [23], and are more likely to switch doctors because of perceived differential treatment because of their weight [24]. Furthermore, perceived weight stigma during medical visits is associated with worsened provider-patient relationships and adherence [25], lower perceived physician empathy [26], and intentions to avoid future medical appointments [27]. Recent research highlights the roles of both experienced weight stigma and internalized weight bias (e.g., applying negative weight stereotypes to oneself and engaging in self-devaluation based on one’s weight) in healthcare avoidance, which explain the relationship between BMI and healthcare avoidance through body-related shame and guilt, and healthcare stress [28]. Given emerging evidence that higher weight bias internalization (WBI) is associated with weight stigma experienced from healthcare providers [29], these findings suggest the importance of studying both people’s experiences of weight stigma in healthcare, and the ways in which internalizing weight stigma may affect their healthcare behaviors or experiences.

Outside of the U.S., studies in other countries have begun to document negative implications of weight stigma for healthcare [30]. For example, recent experimental studies from Australia show evidence of weight bias in health professionals’ treatment decision-making for patients with higher weight [31], and that stigmatizing discussions about weight during doctor-patient interactions reduce patient motivation and compliance [32]. Australian studies have also documented associations between weight stigma and lower engagement with healthcare providers [33], and reported weight stigma across different medical disciplines [34,35]. Likewise, research has documented the presence of weight stigma among healthcare providers in countries like Canada [36–39], France [40], Germany [41–44], and the UK [45], where patient reports of weight stigma and self-reported attitudes of healthcare providers have been examined. However, links between weight stigma and healthcare experiences are less understood in these Western nations, and the different samples, measures, and methodologies (e.g., qualitative vs quantitative) used across these studies make it difficult to conduct comparisons in this emerging literature.

Furthermore, despite research documenting the presence of weight stigma in different parts of the world, cross-country comparisons are lacking in this field of study. This is especially evident with the absence of multinational research examining the implications of weight stigma for healthcare. A 2020 international consensus statement calling for the elimination of weight stigma (supported by more than 100 medical and scientific organizations worldwide) illustrates widespread recognition of weight stigma and its harmful health consequences, including the priority to address weight within the medical community [46]. We have recently begun to address this gap by conducting a multinational study with six countries to compare the nature, extent, and weight-related correlates of experienced weight stigma and internalized weight bias [47,48]. However, to date, no research has yet examined or compared weight stigma in the context of healthcare across different countries. Collective efforts to address weight stigma necessitate broad-scale investigation and cross-country comparisons to advance knowledge of how weight stigma affects healthcare experiences and to identify intervention targets that can help improve quality of healthcare for people with higher weight. As societal and cultural features of different countries may either temper or worsen implications of weight stigma for healthcare, it is important to begin to identify similarities and variations across countries that can inform broader stigma-reduction initiatives.

To begin to address this gap in the literature, the present study aimed to advance knowledge of the associations between weight stigma and healthcare across six Western countries. Using a multinational sample of adults enrolled in an internationally available weight management program, we systematically compared associations between experienced weight stigma, internalized weight bias, and healthcare indices including perceived quality of healthcare, avoidance or delay of medical care, relational experiences with healthcare providers, and weight stigma from doctors. While comparisons across countries were exploratory, we predicted that weight stigma would be associated with adverse healthcare indices across countries.

## Materials and methods

### Participants and procedure

Using data from a larger study examining various aspects of weight stigma in an international sample [47,48], this study focuses on the healthcare experiences of adults from six countries: Australia, Canada, France, Germany, the United Kingdom (UK), and the United States (US). Participants were recruited from enrollees in an internationally available weight management program (WW, formerly Weight Watchers). WW is a validated behavioral weight management program that focuses on healthy habits related to food, activity, and mindset and has been proven effective in multiple randomized controlled trials [49–51]. Individuals at least 18 years of age who had been WW members for a minimum of three months were eligible to participate in the study, which was advertised as a survey to learn about people’s experiences regarding body weight and health, including social experiences and challenges. All study protocols were approved by the institutional review board at the University of Connecticut (Protocol #X17-094).

Participants completed an identical, online questionnaire in the dominant language of their country. Language translation (and back translation) for the survey into French and German was completed by a professional translation services company [52]. Before data collection commenced, surveys were first pilot-tested in each country with small samples to assess survey comprehension. Data collection occurred during the time period of May 2020 to July 2020. Each week, email invitations to the study were sent to a subset of randomly selected WW members in each country (ranging from 4000–33,000 members, mean = 23,474). Participants provided consent in the online survey prior to completing questionnaires. Of those who entered the survey website (*n* = 23,415), 8.0% were ineligible (i.e., declined to consent, were under 18 years old, did not indicate WW program involvement, WW member for less than 3 months, did not complete eligibility questions), and 2.8% who did not indicate residence in one of the six countries (e.g., Singapore) were excluded. After an additional 6,875 individuals were excluded who completed less than fifty percent of the questionnaire and/or did not report plausible key variables (i.e., height and weight, sex, level of education, weight stigma questions), the final analytic sample consisted of 13,996 adults (Australia = 1245, Canada = 2708, France = 2510, Germany = 2613, UK = 2305, US = 2615). Response rates within each country were as follows: 3.8%, Australia; 5.3%, Canada; 5.9%, France; 4.4%, Germany; 4.2% UK; 4.9% US.

Participants in the analytic sample ranged in age from 18 to 89 years (*M =* 47.3–56.9 years, *SD* = 10.7–12.9), predominantly identified as White (range = 91–97%), and female (range = 94–97%) across countries. Participants had an average BMI of 30.5 (*SD* = 6.7), although the mean BMI of participants in France (29.3) was slightly lower than the other five countries. When considering weight status categories, most participants in each country had a BMI≥30 (38–48% in each country: Australia = 594, Canada = 1240, France = 960, Germany = 1209, UK = 1051, US = 1212) or a BMI ranging from 25–29.9 (33–41% in each country: Australia = 457, Canada = 902, France = 1031, Germany = 942, UK = 794, US = 850); less prevalent across countries were participants who had a BMI ranging from 18.5–24.9 (16–21% in each country: Australia = 194, Canada = 556, France = 516, Germany = 461, UK = 457, US = 547), and BMI<18.5 (less than 1% in each country: Australia = 0, Canada = 10, France = 3, Germany = 1, UK = 3, US = 6).

### Measures

#### Healthcare experiences

Participants responded to nine items assessing multiple aspects of healthcare experiences and behaviors, both generally and in specific reference to the past 12 months. All items were derived from previously established and published measures. To assess *general healthcare avoidance*, participants were asked several questions from the Health Information National Trends Survey (HINTS) [53–55], including whether or not they avoid visiting their doctor even when they suspect they should (0 = *Not true*, 1 = *True*), and their agreement with the statement, “I avoid seeing my doctor because I feel uncomfortable when my body is being examined”; responses on a 4-point scale were reverse coded such that higher values reflect greater agreement (i.e., 1 = *Strongly disagree*– 4 = *Strongly agree*). Frequency of obtaining regular checkups was assessed with a single item [56] (i.e. “How often do you obtain regular checkups? [e.g., an annual physical exam and/or dental exam]”) rated on a 5-point scale (1 = *Never* to 5 = *Always*).

To assess more proximal healthcare experiences, participants indicated whether or not there was a time in the past 12 months when they needed medical care (0 = *No*, 1 = *Yes*), as well as whether they delayed or did not get the care they thought they needed (0 = *No*, 1 = *Yes*) [57,58].

Additionally, three items assessed quality of *relational experiences with doctors*: 1) “During the past 12 months, how often did doctors or other health providers listen carefully to you?” [59], 2) “During the past 12 months, how often did doctors or other health providers show respect for what you had to say?” [59], 3) “In the last 12 months, did you ever feel that a doctor judged you because of your weight?” [60]). A final item assessed perceived *quality of healthcare* received (“Overall, how would you rate the quality of health care you received in the past 12 months?”) [53]. Whereas the relational experience items were assessed on a 4-point scale, a 5-point scale was used to measure quality of healthcare received. The items were reverse coded, such that higher values reflect more careful listening and respect by doctors, more doctor judgment (i.e., 1 = *Never* to 4 = *Always*) and perceptions of greater quality of received healthcare (i.e., 1 = *Poor* to 5 = *Excellent*) in the last year.

#### Experienced weight stigma

Three yes/no questions assessed previous history of experienced stigma, asking participants “Have you ever been [teased / treated unfairly / discriminated against] because of your weight? [61]. A dichotomous indicator was created to distinguish individuals who responded “yes” to at least one of the items from those who did not endorse any items (i.e., experienced any weight stigma versus none). In addition, those who reported having experienced weight stigma were asked to indicate the frequency of experiencing weight stigma from doctors on a scale from 0 (*Never*) to 3 (*Multiple times*).

#### Internalized weight bias

Participants responded to the 10-item Modified Weight Bias Internalization Scale (WBIS-M) [62–64], assessing self-directed blame and negative self-judgement due to body weight as well as internalization of negative weight-based stereotypes (e.g., “My weight is a major way that I judge my value as a person”). Reponses on a 7-point scale (1 = *Strongly disagree* to 7 = *Strongly agree*) were averaged, with higher values indicating greater internalization. Internal consistencies were similarly high across the six countries (0.91–0.93).

#### Covariates

Several participant characteristic variables were included as covariates. Participants reported their age, sex, and educational attainment (coded as college degree or equivalent versus no college degree). As it was not permissible by law to collect participant race/ethnicity in France and Germany, this information was not included as a covariate. Self-reported height and weight were used to calculate participants’ BMI; to be inclusive of diverse body sizes, height, weight, and BMI variables were scrutinized case-by-case and implausible values (e.g., “6 inches”; <15 in each country) were removed in the exclusion of key variables process described above. In addition, each participant reported on the duration of their WW membership (*M*_Australia_ = 2.6 years, *M*_Canada_ = 3.6, *M*_France_ = 1.3, *M*_Germany_ = 2.2, *M*_UK_ = 3.0, *M*_US_ = 3.8) and WW membership type, which included Digital (access to the WW app and online tools), Digital + Workshop (access to WW coach-led workshops- in-person and/or virtual- as well as app/online tools), or Personal Coaching + Digital (individual support from a WW coach in addition to app/online tools). Sample WW membership type breakdown in each country is as follows; 32–61% Digital: Australia = 585, Canada = 951, France = 1088, Germany = 1605, UK = 821, US = 838; 38–67% Digital + Workshop: Australia = 605, Canada = 1739, France = 1421, Germany = 986, UK = 1448, US = 1760; 0–4% Personal Coaching + Digital: Australia = 55, Canada = 18, France = 1, Germany = 22, UK = 36, US = 17. Personal Coaching + Digital membership type was removed as a covariate in France regression and mediation models due to low prevalence.

#### Analytic plan

Sample characteristics related to demographics, anthropometrics, and weight stigma experienced from doctors are reported first. Descriptive healthcare experience information and unadjusted associations with stigma (experienced and internalized) are subsequently provided. Between-country differences in healthcare experiences overall, and as a function of experienced weight stigma, were assessed using chi-square tests and one-way analyses of variance (ANOVA), respectively. Bivariate correlations were used to measure the strength of associations between WBIS and each of the (continuous) healthcare indicators within each country.

Next, we present regression models examining links between stigma (experienced and internalized) and healthcare experiences, over and above demographics, BMI, and WW variables. Although internalized weight bias is higher among individuals who have, versus have not, experienced weight stigma [*t*(13264.69) = -42.86, *p* < .001; a distinction retained across all countries with all *p*’s < .001], the constructs are conceptually distinct. Whereas linear regression was used to predict continuous outcomes (e.g., frequency of obtaining regular checkups), logit models were constructed to predict general avoidance of doctor, and delaying and/or not getting needed healthcare in the last 12 months. Finally, we turn to mediation models that examine the indirect effect of experienced stigma on the healthcare indicators through internalized weight bias; a montecarlo integration algorithm was used for the two binary outcome variables (i.e., general avoidance of doctor, avoidance of needed healthcare). Regression and mediation analyses accounted for age, sex (male, female), educational attainment (college degree or equivalent, no college degree or equivalent), BMI, WW membership duration and membership type (Digital, Digital + Workshop, Personal Coaching + Digital). Participants who identified as “other” sex were excluded from the regression and mediation analyses given low prevalence across countries (*n* = 0–6). A log transformation was used to correct for non-normality in BMI and WW membership duration; age, BMI, and WW membership duration were centered within each country. Descriptive analyses were performed in SPSS (version 27), while regression analyses and mediation models were conducted in Mplus. Missing data was handled with listwise deletion. To reduce the likelihood of Type I error (given the large sample size), statistical significance was set at p≤.001 [65,66]. Ninety-nine percent confidence intervals are reported for the indirect effect estimates.

## Results

Table 1 displays frequency of experiencing weight stigma from doctors. Weight stigma from doctors was reported by two-thirds (66.6%) of participants across countries who indicated experiencing any weight stigma (i.e., those not selecting “never” to any type of stigma, independent of source). Prevalence of weight stigma from doctors was more common in Germany (73.5%) compared to all other countries, with the exception of the US.

**Table 1:** Frequency of experiencing weight stigma from doctors.

	Total sample	Australia	Canada	France	Germany	United Kingdom	United States
	% (N)	% (N)	% (N)	% (N)	% (N)	% (N)	% (N)
*Frequency of experiencing weight stigma from a doctor*:							
Multiple times	19.8 (1570)	20.1 (137)^a,c^	20.7 (336)^a,c^	11.6 (157)^b^	21.3 (303)^a,c^	19.6 (256)^a^	24.8 (381)^c^
More than once	26.4 (2087)	23.3 (159)^a,b^	24.8 (403)^a,b^	28.1 (379)^a,b^	29.7 (422)^a^	24.1 (314)^b^	26.7 (410)^a,b^
Once	20.4 (1611)	19.1 (130)^a,b^	19.3 (313)^a,b^	23.7 (320)^a^	22.4 (318)^a,b^	19.2 (250)^a,b^	18.2 (280)^b^
Never	33.4 (2647)	37.4 (255)^a^	35.2 (572)^a,c^	36.5 (492)^a^	26.5 (377)^b^	37.2 (485)^a^	30.3 (466)^b,c^

*Note*. Item only administered to participants who reported at least one experience of weight stigma. Values within the same row not sharing the same subscript letter are significantly different from each other at *p* ≤ .001.

### Descriptive healthcare information and associations with stigma

On average, 29% of participants across countries avoided visiting their doctor even when they suspected they should. Between-country differences emerged in healthcare avoidance, *χ*^*2*^(5) = 182.34, *p* < .001. Follow-up comparisons indicated that prevalence of healthcare avoidance was significantly higher in the UK (39%) versus other countries. Healthcare avoidance was more common in Germany (32%) and France (30%) compared to Canada (24%) and the US (24%).

The majority (70%; n = 9592) of participants indicated needing medical care in the past 12 months. Between-country differences were revealed [*χ*^*2*^(5) = 122.17, *p* < .001], with follow-up comparisons indicating that need of medical care in the last year was significantly higher in Australia (79%; n = 968) compared to all other countries, with the exception of Germany (74%; n = 1894). In addition, medical need in the past year was more common in Germany compared to France (69%; n = 1683), Canada (67%; n = 1774), as well as the UK (64%; n = 1451) which was in turn significantly lower than France and the US (71%; n = 1822). Chi-square tests revealed that reports of needing medical care in the last year were similar between males and females within each country [Australia: *χ*^*2*^(1) = 1.32, *p* = .251, Canada: *χ*^*2*^(1) = 9.07, *p* = .003, France: *χ*^*2*^(1) = 1.97, *p* = .160, Germany: *χ*^*2*^(1) = 0.01, *p* = .921, UK: *χ*^*2*^(1) = 0.29, *p* = .590, US: *χ*^*2*^(1) = 1.73, *p* = .189]. Within-country independent samples t-tests indicated no BMI differences between individuals who reported needing medical care in the past 12 months in the US [*t*(1530.86) = -2.26, *p* = .024], Germany [*t*(2552) = -1.74, *p* = .082], and France [*t*(1581.09) = -2.97, *p* = .003]; however, in Australia [*t*(434.55) = -4.88, *p* < .001], Canada [*t*(1835.39) = -4.26, *p* < .001], and the UK [*t*(1777.83) = -4.48, *p* < .001], higher BMI was documented among those who needed medical care in the past year.

Among those who reported needing medical care in the past year, 22% overall indicated delaying or not getting the care they thought they needed. Significant between-country differences emerged [*χ*^*2*^(5) = 90.23, p < .001], such that prevalence of avoidance of healthcare was significantly lower in the US (16%) compared to all other countries. In addition, avoidance was less common in Canada (20%) relative to France (25%), and the UK (28%) which in turn was significantly greater than Germany (21%).

Table 2 details descriptive information and across country comparisons for the continuous healthcare indicators, including both general healthcare experiences, as well as those pertaining to the past 12 months (examined only among individuals who indicated needing medical care in the last year). Within-country t-tests revealed that across each of the six nations, individuals who had experienced weight stigma reported obtaining less frequent regular checkups (all *p*’s <0.001) and greater healthcare avoidance due to feeling uncomfortable with body examination (all *p*’s <0.001) compared to those who had not experienced weight stigma. In addition, across all countries, individuals who had experienced weight stigma reported that during the past 12 months their doctors less frequently listened carefully to them (all *p*’s <0.001), less frequently respected what they had to say (all *p*’s <0.001), and judged them more frequently because of their weight (all *p*’s <0.001) relative to those who had not experienced weight stigma. Furthermore, in each country, perceived quality of healthcare received in the last year was significantly lower among individuals who had, versus had not, experienced weight stigma (all *p*’s ≤0.001). Taken together, despite across-country variation in mean levels of the healthcare indicators, experienced weight stigma functioned with considerable consistently in each country–relating more negatively to overall and recent healthcare experiences.

**Table 2:** Descriptive information about healthcare experiences, stratified by country and experienced weight stigma.

Healthcare Experiences	Total sample	Australia	Canada	France	Germany	United Kingdom	United States		
	M (SD)	M (SD)	M (SD)	M (SD)	M (SD)	M (SD)	M (SD)	*F*	p
General experiences									
*How often do you obtain regular checkups*?^1^	3.96 (1.19)	3.68^a^ (1.22)	4.15^b^ (1.07)	3.68^a^ (1.21)	4.11^b^ (1.11)	3.52^c^ (1.38)	4.38^d^ (0.96)	199.59	< .001
Any weight stigma	3.86 (1.22)	3.47 (1.26)	4.07 (1.10)	3.59 (1.23)	4.02 (1.14)	3.43 (1.38)	4.27 (1.03)		
No weight stigma	4.09 (1.14)	3.93 (1.13)	4.29 (1.01)	3.79 (1.17)	4.23 (1.06)	3.66 (1.37)	4.53 (0.82)		
*I avoid seeing my doctor because I feel uncomfortable when my body is being examined*.^2^	1.80 (0.99)	1.89^a^ (1.04)	1.77^b^ (1.00)	1.72^b^ (0.90)	1.78^b^ (0.96)	1.97^a^ (1.06)	1.74^b^ (0.99)	21.19	< .001
Any weight stigma	1.99 (1.05)	2.11 (1.08)	1.96 (1.06)	1.86 (0.94)	1.97 (1.03)	2.21 (1.11)	1.93 (1.05)		
No weight stigma	1.53 (0.83)	1.61 (0.90)	1.48 (0.81)	1.54 (0.80)	1.52 (0.81)	1.64 (0.89)	1.45 (0.81)		
Experiences during past 12 months^3^									
*How often did doctors or other health providers listen carefully to you*?^4^	3.27 (0.77)	3.32^adf^ (0.74)	3.36^ab^ (0.74)	3.17^ce^ (0.75)	3.25^cd^ (0.78)	3.14^e^ (0.84)	3.36^bf^ (0.72)	24.11	< .001
Any weight stigma	3.18 (0.79)	3.23 (0.77)	3.26 (0.77)	3.10 (0.76)	3.19 (0.78)	3.01 (0.86)	3.26 (0.75)		
No weight stigma	3.41 (0.71)	3.48 (0.67)	3.54 (0.64)	3.27 (0.72)	3.34 (0.77)	3.36 (0.75)	3.52 (0.61)		
*How often did doctors or other health providers show respect for what you had to say*?^4^	3.31 (0.76)	3.40^acf^ (0.71)	3.45^ab^ (0.70)	3.35^c^ (0.71)	3.02^d^ (0.80)	3.26^e^ (0.82)	3.45^bf^ (0.68)	86.40	< .001
Any weight stigma	3.22 (0.78)	3.28 (0.74)	3.36 (0.74)	3.27 (0.74)	2.94 (0.79)	3.13 (0.85)	3.35 (0.72)		
No weight stigma	3.45 (0.70)	3.58 (0.63)	3.62 (0.60)	3.45 (0.66)	3.13 (0.79)	3.46 (0.72)	3.62 (0.56)		
*Did you ever feel that a doctor judged you because of your weight*?^4^	1.43 (0.72)	1.45^ac^ (0.75)	1.40^ab^ (0.72)	1.46^ac^ (0.69)	1.33^b^ (0.61)	1.53^c^ (0.84)	1.46^ac^ (0.74)	14.61	< .001
Any weight stigma	1.59 (0.81)	1.62 (0.84)	1.54 (0.81)	1.59 (0.75)	1.47 (0.69)	1.74 (0.95)	1.63 (0.83)		
No weight stigma	1.18 (0.46)	1.18 (0.48)	1.13 (0.41)	1.28 (0.54)	1.14 (0.42)	1.20 (0.46)	1.18 (0.44)		
*Perceived quality of received healthcare*^5^	3.98 (0.97)	4.16^a^ (0.92)	4.20^a^ (0.93)	3.89^b^ (0.82)	3.59^c^ (1.01)	3.92^b^ (1.05)	4.19^a^ (0.89)	113.66	< .001
Any weight stigma	3.89 (0.99)	4.04 (0.96)	4.09 (0.95)	3.84 (0.83)	3.51 (1.02)	3.79 (1.09)	4.07 (0.93)		
No weight stigma	4.11 (0.92)	4.35 (0.83)	4.40 (0.84)	3.97 (0.82)	3.69 (0.98)	4.13 (0.95)	4.38 (0.80)		

*Note*. Values within the same row not sharing the same letter are significantly different from each other at *p* ≤ .001.

^1^Response options ranged from 1 (*Never*) to 5 (*Always*).

^2^Response options ranged from 1 (*Strongly disagree*) to 4 (*Strongly agree*).

^3^Descriptives calculated only among individuals who indicated needing medical care in the last year.

^4^Response options ranged from 1 (*Never*) to 4 (*Always*).

^5^Response options ranged from 1 (*Poor*) to 5 (*Excellent*).

**Fig 1:**
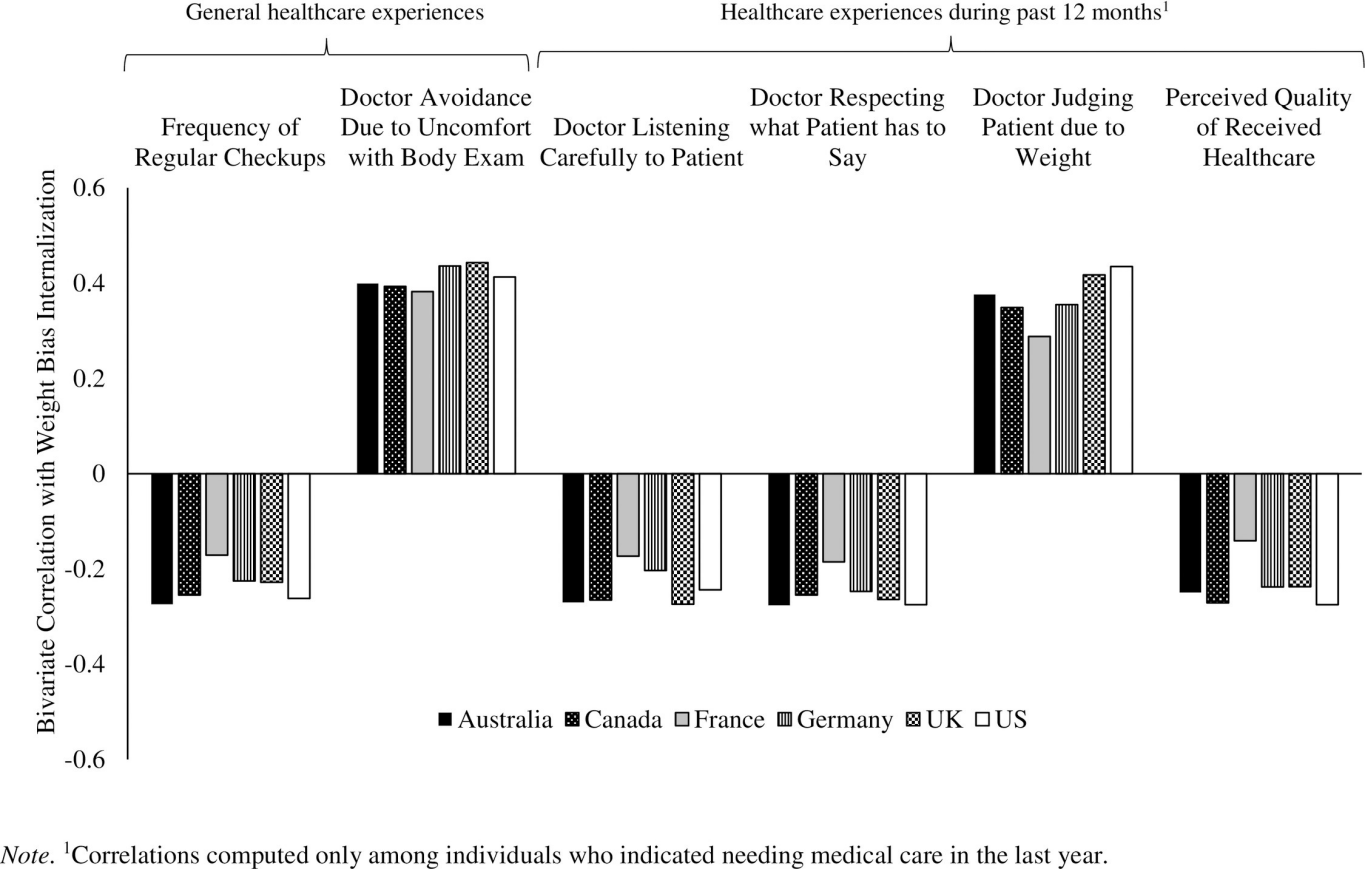
Bivariate correlations between weight bias internalization and (continuous) healthcare experience indicators.

To assess the strength of associations between weight bias internalization (WBI) and each of the (continuous) healthcare indicators, within-country unadjusted bivariate correlations were computed. Weight bias internalization was most strongly associated with general healthcare avoidance due to feeling uncomfortable with a bodily exam (*r*’s = .38 to .44), followed by judgement by doctor due to weight in the last 12 months (*r*’s = .29 to .44) (Fig 1). Small negative associations emerged between WBI and frequency of regular checkups (*r*’s = -.17 to -.27), as well as doctor listening carefully to patient (*r*’s = -.17 to -.27), doctor respecting what patient has to say (*r*’s = -.19 to -.28), and perceived quality of care (*r*’s = -.14 to -.28) in the past year.

### Regression models predicting healthcare experiences

Table 3 presents associations between weight stigma (experienced and internalized) and the healthcare indicators in each country above and beyond the demographic, anthropometric and weight management covariates. After accounting for the covariates and experienced weight stigma, WBI was a significant predictor of each healthcare indicator. Specifically, individuals reporting higher levels of WBI in each country indicated greater avoidance of seeing one’s doctor due to being uncomfortable with the body exam, increased perceived judgment from doctors due to body weight in the last 12 months, and greater likelihood of healthcare avoidance in general and when healthcare was needed. In addition, reported frequency of regularly obtaining checkups, perceived careful listening as well as respect from doctors, and quality of received healthcare in the last 12 months were all negatively associated with WBI in each country.

**Table 3:** Associations between weight stigma (experienced and internalized) and healthcare experience indicators.

	Australia		Canada		France		Germany		United Kingdom		United States	
	β	*p*	β	*p*	β	*p*	β	*p*	β	*p*	β	*p*
*Frequency of obtaining regular checkups*												
Experienced Weight Stigma	-0.17	.006	-0.03	.413	-0.03	.567	-0.01	.746	0.05	.293	-0.08	.056
Internalized Weight Bias	**-0.19**	< .001	**-0.21**	< .001	**-0.14**	< .001	**-0.20**	< .001	**-0.18**	< .001	**-0.18**	< .001
BMI	0.03	.436	0.01	.543	0.02	.415	-0.03	.124	-0.03	.211	-0.02	.330
*Doctor avoidance due to feeling uncomfortable with body exam*												
Experienced Weight Stigma	**0.19**	.001	**0.25**	< .001	0.12	.003	**0.16**	< .001	**0.22**	< .001	**0.21**	< .001
Internalized Weight Bias	**0.34**	< .001	**0.33**	< .001	**0.34**	< .001	**0.38**	< .001	**0.37**	< .001	**0.34**	< .001
BMI	0.04	.222	0.05	.018	0.06	.007	0.06	.005	**0.08**	< .001	0.04	.089
*Doctor listening carefully to patient* *in last 12 months*^1^												
Experienced Weight Stigma	-0.16	.024	**-0.24**	< .001	-0.10	.059	-0.06	.203	**-0.24**	< .001	**-0.23**	< .001
Internalized Weight Bias	**-0.24**	< .001	**-0.23**	< .001	**-0.13**	< .001	**-0.20**	< .001	**-0.22**	< .001	**-0.19**	< .001
BMI	0.01	.887	0.02	.549	-0.04	.182	0.02	.459	0.00	.954	0.03	.250
*Doctor respecting what patient has to say* *in last 12 months*^1^												
Experienced Weight Stigma	**-0.26**	< .001	**-0.25**	< .001	-0.12	.017	-0.09	.075	**-0.22**	< .001	**-0.22**	< .001
Internalized Weight Bias	**-0.24**	< .001	**-0.22**	< .001	**-0.14**	< .001	**-0.24**	< .001	**-0.19**	< .001	**-0.23**	< .001
BMI	0.03	.428	0.01	.680	-0.04	.103	0.02	.401	-0.05	.107	0.02	.468
*Doctor judging patient due to weight* *in last 12 months*^1^												
Experienced Weight Stigma	**0.24**	< .001	**0.29**	< .001	**0.16**	.001	**0.26**	< .001	**0.28**	< .001	**0.25**	< .001
Internalized Weight Bias	**0.19**	< .001	**0.18**	< .001	**0.16**	< .001	**0.22**	< .001	**0.23**	< .001	**0.29**	< .001
BMI	**0.28**	< .001	**0.26**	< .001	**0.28**	< .001	**0.20**	< .001	**0.29**	< .001	**0.21**	< .001
*Perceived quality of received healthcare* *in last 12 months*^1^												
Experienced Weight Stigma	-0.17	.010	**-0.19**	< .001	-0.04	.419	-0.04	.397	-0.16	.004	**-0.16**	.001
Internalized Weight Bias	**-0.19**	< .001	**-0.24**	< .001	**-0.10**	.001	**-0.25**	< .001	-**0.20**	< .001	**-0.22**	< .001
BMI	0.00	.919	0.02	.522	-0.08	.002	0.03	.214	-0.01	.785	0.00	.884
	*OR*	*p*	*OR*	*p*	*OR*	*p*	*OR*	*p*	*OR*	*p*	*OR*	*p*
*General avoidance of doctor*												
Experienced Weight Stigma	**1.98**	< .001	**1.52**	< .001	1.10	.356	1.06	.577	1.22	.055	1.22	.086
Internalized Weight Bias	**1.43**	< .001	**1.47**	< .001	**1.33**	< .001	**1.38**	< .001	**1.40**	< .001	**1.49**	< .001
BMI	0.75	.479	1.45	.140	1.20	.527	1.45	.145	1.37	.169	1.69	.046
*Delayed or did not get needed healthcare* *in last 12 months*^1^												
Experienced Weight Stigma	1.61	.013	1.49	.008	1.09	.507	1.21	.139	1.34	.042	1.49	.017
Internalized Weight Bias	**1.44**	< .001	**1.38**	< .001	**1.28**	< .001	**1.31**	< .001	**1.58**	< .001	**1.47**	< .001
BMI	1.30	.561	1.17	.622	2.67	.005	1.15	.667	0.74	.357	1.46	.278

*Note*. Regression models run separately for each country and separately for each healthcare outcome variable. In addition to BMI, covariates include age, sex, educational attainment, WW membership duration and membership type. Boldface indicates statistical significance.

^1^Estimated models include only individuals who indicated needing medical care in the last year. (A similar pattern of results emerged when examining the associations among the full sample).

After accounting for WBI and the set of covariates, direct associations between experienced weight stigma and two of the healthcare indicators (i.e., frequency of obtaining regular checkups in general, and delaying or not getting needed healthcare in the last 12 months) were non-significant, consistently across all six countries. However, individuals who reported experienced weight stigma indicated more frequent judgment from doctors due to their weight in the last 12 months across all countries. Differential associations emerged across countries when considering perceived quality of received healthcare, respect and careful listening from doctors in the last 12 months, as well as healthcare avoidance due to being uncomfortable with the body examination and general avoidance of seeing one’s doctor (see Table 3). For example, individuals who indicated having experienced at least one instance of weight stigma from any source (versus none) reported a greater avoidance due to feeling uncomfortable with one’s body examination in all countries, except in France. In addition, while experienced weight stigma was associated with a greater odds of general healthcare avoidance in Australia (*OR* = 1.98, *p* < .001) and Canada (*OR* = 1.52, *p* < .001), the direct association was non-significant in all other countries.

### Mediation models examining indirect effects of experienced stigma

To test how experienced stigma might indirectly contribute to healthcare experiences, we examined the link between experienced weight stigma on each of the eight healthcare indicators through internalized weight bias within the six countries. Above and beyond the demographic, anthropometric and weight management covariates, the indirect path from experienced weight stigma to healthcare experiences through internalized weight bias was significant for all eight outcomes, in all six countries (S1–S8 Figs). Individuals who had, versus had not, experienced weight stigma reported higher levels of internalized weight bias, which in turn was related to reduced frequency of obtaining regular checkups [indirect effects: Australia = -0.09, Canada = -0.08, France = -0.06, Germany = -0.09, UK = -0.09, US = -0.08], greater doctor avoidance due to feeling uncomfortable when body is being examined [indirect effects: Australia = 0.16, Canada = 0.13, France = 0.14, Germany = 0.18, UK = 0.18, US = 0.15], and greater likelihood of general doctor avoidance [indirect effects: Australia = 0.13, Canada = 0.12, France = 0.09, Germany = 0.11, UK = 0.13, US = 0.13].

A similar pattern of indirect effects emerged when considering each of the indicators of healthcare behaviors and experiences in the past 12 months (examined only among individuals who indicated needing medical care in the last year). Specifically, experienced weight stigma was associated with higher levels of internalization, which in turn was related to lower perceived quality of healthcare received in the last 12 months [indirect effects: Australia = -0.09, Canada = -0.09, France = -0.05, Germany = -0.11, UK = -0.09, US = -0.10], greater likelihood of avoidance of needed healthcare in the past year [indirect effects: Australia = 0.13, Canada = 0.10, France = 0.08, Germany = 0.09, UK = 0.16, US = 0.13], and poorer patient-provider relationships in the last 12 months as indicated by perceptions of less careful listening [indirect effects: Australia = -0.12, Canada = -0.09, France = -0.06, Germany = -0.09, UK = -0.10, US = -0.09] and less respect [indirect effects: Australia = -0.12, Canada = -0.09, France = -0.07, Germany = -0.11, UK = -0.09, US = -0.10] by doctors, and more doctor judgment [indirect effects: Australia = 0.09, Canada = 0.07, France = 0.08, Germany = 0.10, UK = 0.11, US = 0.13].

Taken together, WBI was consistently linked to the healthcare indices across the six countries. Despite variation in the direct associations between experienced weight stigma and healthcare experiences, the indirect effects through internalized weight bias were consistent across each of the healthcare indicators and across each of the six countries. Further, the near entirety of non-significant associations between BMI and the healthcare indicators underscores the critical role that social psychological processes play in healthcare behaviors.

## Discussion

There is much that is unknown about the relationship between internalized weight stigma and healthcare, and there is little published work on this relationship in countries outside of the US. Our study makes a significant contribution as the first multinational comparison of links between weight stigma and healthcare across different countries, using identical measures and comparable samples. Our findings illustrate considerable consistency in associations between weight stigma and adverse healthcare indices across each of the six countries investigated. Compared to individuals with no history of weight stigma, unadjusted/raw associations revealed that participants across countries who had experienced weight stigma reported obtaining less frequent regular medical checkups, greater healthcare avoidance due to feeling uncomfortable with their body being examined, more frequent judgment from doctors due to their weight, worse quality of their recent healthcare experiences, and reported that their doctors less frequently listened carefully to them and less frequently respected what they had to say. This consistent pattern of results highlights a concerning role of weight stigma in healthcare experiences in multiple countries, regardless of between-country variation in overall levels of reported medical need and healthcare delay or avoidance.

Underscoring healthcare as a critical context for negative weight-related judgement, devaluation and unfair treatment, our findings shed light on the universality of weight stigma within the healthcare setting. Across all countries in the present investigation, perceived weight stigmatization in the healthcare environment was a common phenomenon. For example, among participants who had a history of experiencing weight stigma, on average two-thirds across countries reported being stigmatized about their weight from a doctor. This finding is somewhat similar to prevalence estimates among US samples of adults engaged in weight management who have reported experiencing weight stigma from healthcare professionals [19,20]. Given that the majority (70%) of participants across the six countries in our study indicated needing medical care in the past 12 months, the commonality of weight stigma from doctors reported by participants represents a significant public health concern.

Importantly, our findings highlight the significant role of weight bias internalization (WBI) in healthcare experiences for people engaged in weight management across different countries, extending initial evidence within a US sample [28]. Indeed, WBI retained unique predictive value for each of the healthcare indicators after accounting for covariate effects (e.g., demographic characteristics, BMI), while direct effects between experienced weight stigma and two of the healthcare indicators (i.e., frequency of obtaining regular checkups, delaying or not getting needed healthcare in the last 12 months) were no longer significant. Specifically, in each of the six countries, participants with higher WBI reported greater avoidance of seeing one’s doctor due to being uncomfortable with the body exam, increased perceived judgment from their doctor due to body weight, increased likelihood of healthcare avoidance (both in general and when medical attention was needed), lower frequency of regularly obtaining checkups, less frequent listening and respect from doctors, and worse quality of healthcare in the last 12 months. Moreover, our mediation analyses findings imply that internalized weight bias accounts in part for compromised healthcare experiences of individuals who have been teased, discriminated against, and/or treated unfairly because of their weight. In other words, experiencing weight stigma contributes to greater internalization of weight bias, which in turn relates to poorer healthcare experiences. Notably, the indirect effects of experienced weight stigma to healthcare behaviors and experiences through internalized weight bias were consistent across all healthcare indices, and across all six countries.

Together, these findings parallel emerging US studies implicating both experienced weight stigma [22,23,25,28] and WBI [22,28] as factors associated with poorer patient-provider relationships and reduced quality of care, both in community samples [23,28] and among samples of people with medical diagnoses like type 2 diabetes [22] and hypothyroidism [25]. Given limited research attention to these relationships in treatment-seeking and general population samples, our findings underscore the need for increased research studies to assess the effects of WBI on healthcare utilization, quality, and patient outcomes, which have not yet been adequately examined in the literature. In particular, prospective and experimental studies will provide important insights into whether adverse healthcare experiences contribute to WBI or vice versa.

Collectively, our study offers several key insights. First, our results indicate that WBI is a consistent correlate of healthcare indices for people engaged in weight management across different countries, and may play a more proximal role in contributing to adverse healthcare experiences than an individual’s history of experienced weight stigma. Recent studies in North America, Europe, and Australia have found that WBI uniquely contributes to psychological distress, disordered eating behaviors, and adverse physical health indices, over and above other demographic and anthropomorphic covariates [67]. Our findings add to this evidence suggesting that the negative implications of WBI extend to healthcare experiences among people engaged in weight management. Given that WBI has received limited research attention in the context of healthcare, this should be a priority for future research. Second, most associations between participants’ BMI and healthcare indicators were non-significant across the six countries in our study, emphasizing the importance of stigma processes in healthcare behaviors, rather than body weight per se. WBI can adversely affect health and wellbeing for people of diverse body sizes [67]; thus, rather than limiting research attention to links between obesity and healthcare, our findings underscore the need to focus on stigma-related risk factors, mechanisms, and barriers that impact healthcare, independent of BMI. Obtaining regular care, minimizing delays in healthcare, and promoting effective provider-patient communication are vital to early detection and effective treatment of a number of chronic diseases [68–70], of which individuals with high weight may be at elevated risk. Given that the challenges and barriers caused by weight stigma within healthcare may exacerbate poor health and treatment outcomes, eliminating weight stigma is an essential step to ensure more effective, stigma-free care and treatment.

### Limitations and strengths

Several limitations are present in this study that should be taken into account in interpreting the findings. The cross-sectional data do not allow for causal conclusions to be made with respect to the relationship between healthcare experiences and weight stigma; there is a need for longitudinal examination of these constructs. Our data relied on self-reported recall of stigma and healthcare experiences; as such, more comprehensive assessment, including data from healthcare records, would be informative. Relatedly, self-report bias may be present given that participants who reported weight stigma (experienced and/or internalized) may be more likely to perceive threat and appraisal social cues more negatively, including treatment from healthcare professionals. Further, not all of the measures used in this study have been validated in every language. For example, the psychometric properties of the Weight Bias Internalization Scale have been evaluated in German populations [71], but not yet in France or many other non-English speaking countries, indicating the need for broader scale validation across countries. The racial/ethnic and gender diversity of our sample was limited primarily to white women, and those of middle age; examination of links between weight stigma and healthcare experiences in more diverse multinational samples is warranted, particularly given evidence that internalization of weight stigma may be different for women and men, and among individuals of different racial/ethnic backgrounds [72]. Our study was also limited to Western countries, and future cross-country research should include other parts of the world where experiences of weight stigma may be different. The low response rate prevents generalization to all WW members and/or those seeking treatment, and our study findings should be interpreted accordingly. Similarly, our study samples may not be representative of individuals with higher weight in general; given that individuals with obesity are more likely to report experiencing discrimination in healthcare than individuals at lower weights [73], additional studies are needed with both treatment-seeking and community samples of people with high weight, including individuals who are not engaged in weight management. Finally, attention checks were not present, and data were collected during the spring/summer of 2020 in the midst of the COVID-19 pandemic, which may have affected the nature of participants’ responses and/or the survey response rate.

Despite these limitations, several aspects of our study strengthen its contribution to the literature. First, in addition to the large sample size, our data provide the first multinational comparison of weight stigma and healthcare, contributing novel insights to the scant cross-cultural research in the weight stigma literature. Second, the use of identical measures and similar samples across countries allows for meaningful comparisons of key variables that have not previously been examined in different countries. Third, the inclusion of simultaneous modeling of both experienced weight stigma and internalized weight bias allow for novel insights about the pathways by which stigma contributes to healthcare experiences (i.e., experienced weight stigma → internalized weight bias → adverse healthcare experiences).

### Conclusions

Our study has clear implications for collective, cross-country initiatives to address weight stigma in the context of healthcare. In addition to prioritizing research to better understand the impact of weight stigma on healthcare experiences, utilization, and patient outcomes, our findings indicate the importance of establishing a healthcare culture free of weight stigma. Increasingly, national organizations in countries like the US [74], Canada [75], the UK [76] as well as Europe [77] have called for initiatives to address weight stigma, including improvements in communication and engagement between health professionals and people with obesity [70]. Most recently, a 2020 international joint consensus called for efforts to reduce weight stigma from multiple stakeholders, including actions from the medical community [46]. Key recommendations from this consensus statement include calling upon professional bodies to facilitate and develop methods to certify knowledge of weight stigma, its harmful effects, and stigma-free practice skills among healthcare providers. Implementing education and training of healthcare professionals and practitioners will be critical to these efforts, and will require increased awareness of personal biases, understanding of the ways in which weight stigma negatively affects health and patient care, and education of strategies to reduce weight stigma in healthcare encounters and clinical practice. As suggested by our study findings, these efforts are needed in multiple countries; thus, collective and collaborative initiatives to address weight stigma should be prioritized.

## Supporting information

S1 Fig Standardized effect estimates of experienced weight stigma on frequency of obtaining regular checkups through internalized weight bias, separately for each country. Covariates included age, sex, educational attainment, BMI, WW membership duration, WW membership type. *p≤.001.
(PDF)

S2 Fig Standardized effect estimates of experienced weight stigma on doctor avoidance (due to feeling uncomfortable with body exam) through internalized weight bias, separately for each country. Covariates included age, sex, educational attainment, BMI, WW membership duration, WW membership type. *p≤.001.
(PDF)

S3 Fig Standardized effect estimates of experienced weight stigma on doctor listening carefully to patient through internalized weight bias, separately for each country. Covariates included age, sex, educational attainment, BMI, WW membership duration, WW membership type. *p≤.001.
(PDF)

S4 Fig Standardized effect estimates of experienced weight stigma on doctor showing respect for what patient has to say through internalized weight bias, separately for each country. Covariates included age, sex, educational attainment, BMI, WW membership duration, WW membership type. *p≤.001.
(PDF)

S5 Fig Standardized effect estimates of experienced weight stigma on perceptions of doctor judging patient due to weight through internalized weight bias, separately for each country. Covariates included age, sex, educational attainment, BMI, WW membership duration, WW membership type. *p≤.001.
(PDF)

S6 Fig Standardized effect estimates of experienced weight stigma on perceived quality of healthcare received in last 12 months through internalized weight bias, separately for each country. Covariates included age, sex, educational attainment, BMI, WW membership duration, WW membership type. *p≤.001.
(PDF)

S7 Fig Standardized effect estimates of experienced weight stigma on general avoidance of doctor through internalized weight bias, separately for each country. Covariates included age, sex, educational attainment, BMI, WW membership duration, WW membership type. *p≤.001.
(PDF)

S8 Fig Standardized effect estimates of experienced weight stigma on avoidance of needed healthcare in last 12 months through internalized weight bias, separately for each country. Covariates included age, sex, educational attainment, BMI, WW membership duration, WW membership type. *p≤.001.
(PDF)
