# Supplementary material for: The roles of experienced and internalized weight stigma in healthcare experiences: Perspectives of adults engaged in weight management across six countries
Source: PLoS One. 2021 Jun 1;16(6):e0251566. doi: 10.1371/journal.pone.0251566 (PMC8168902; doi:10.1371/journal.pone.0251566)
Supplement: S1 Fig — Covariates included age, sex, educational attainment, BMI, WW membership duration, WW membership type. *p≤.001. (PDF) [file pone.0251566.s001.pdf]

Figure 2. Standardized estimates of experienced weight stigma on frequency of obtaining regular checkups through internalized weight bias, separately for each country. Covariates included age, sex, educational attainment, BMI, WW membership duration, WW membership type. $*p \leq .001$.

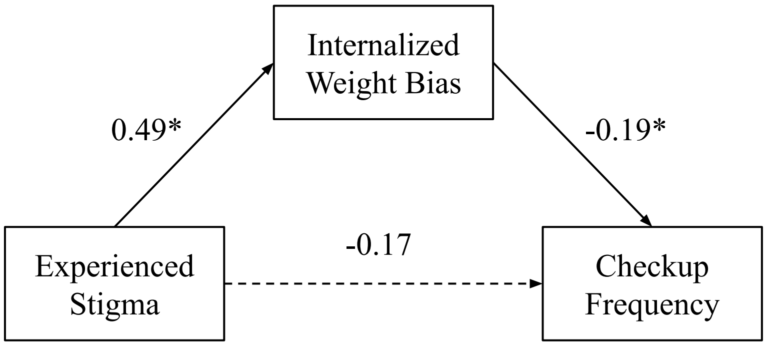

Indirect Effect = -0.09, 99% CI: -0.14 to -0.04

Figure 2a. Indirect effect of experienced stigma, **Australia**

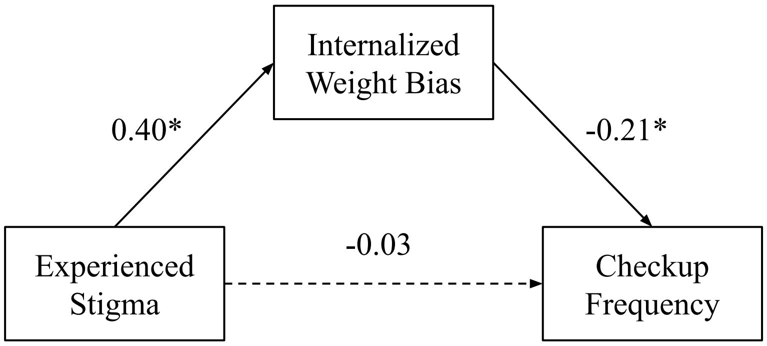

Indirect Effect = -0.08, 99% CI: -0.11 to -0.05

Figure 2b. Indirect effect of experienced stigma, **Canada**

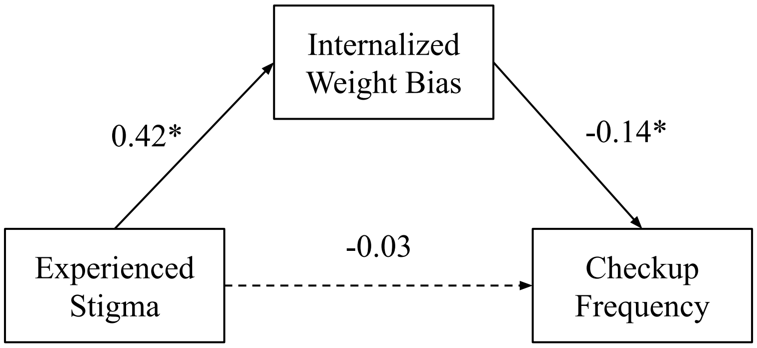

Indirect Effect = -0.06, 99% CI: -0.09 to -0.03

Figure 2c. Indirect effect of experienced stigma, **France**

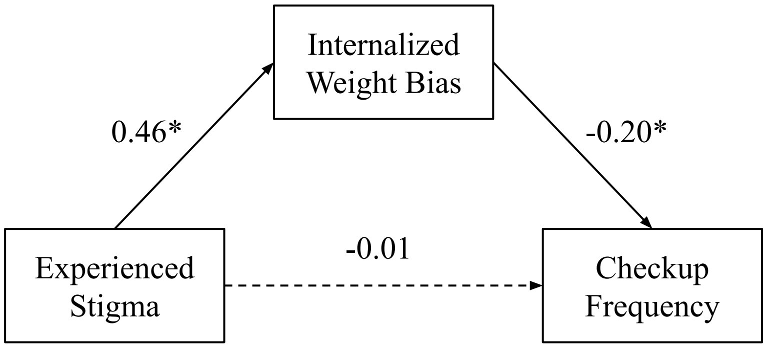

Indirect Effect = -0.09, 99% CI: -0.12 to -0.06

Figure 2d. Indirect effect of experienced stigma, **Germany**

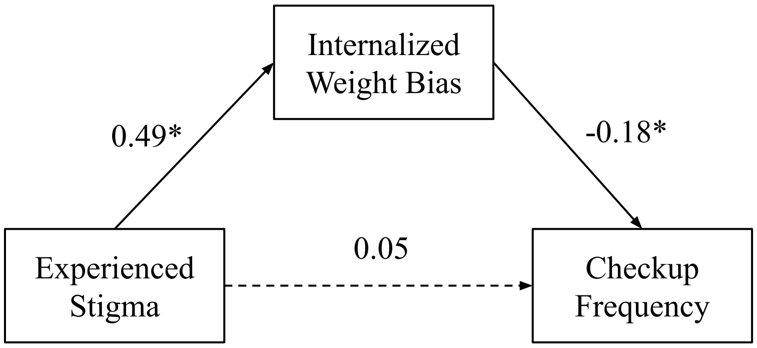

Indirect Effect = -0.09, 99% CI: -0.12 to -0.05

Figure 2e. Indirect effect of experienced stigma, **United Kingdom**

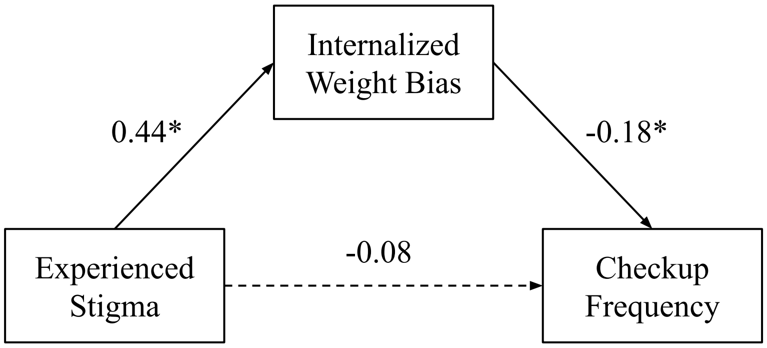

Indirect Effect = -0.08, 99% CI: -0.11 to -0.05

Figure 2f. Indirect effect of experienced stigma, **United States**
