# Supplementary material for: The roles of experienced and internalized weight stigma in healthcare experiences: Perspectives of adults engaged in weight management across six countries
Source: PLoS One. 2021 Jun 1;16(6):e0251566. doi: 10.1371/journal.pone.0251566 (PMC8168902; doi:10.1371/journal.pone.0251566)
Supplement: S2 Fig — Covariates included age, sex, educational attainment, BMI, WW membership duration, WW membership type. *p≤.001. (PDF) [file pone.0251566.s002.pdf]

Figure 3. Standardized effect estimates of experienced weight stigma on doctor avoidance due to feeling uncomfortable with body exam through internalized weight bias, separately for each country. Covariates included age, sex, educational attainment, BMI, WW membership duration, WW membership type. *$p \leq .001$.

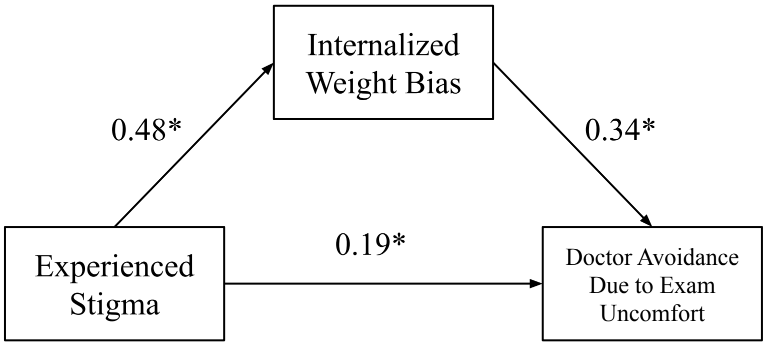

Indirect Effect = 0.16, 99% CI: 0.10 to 0.22

Figure 3a. Indirect effect of experienced stigma, **Australia**

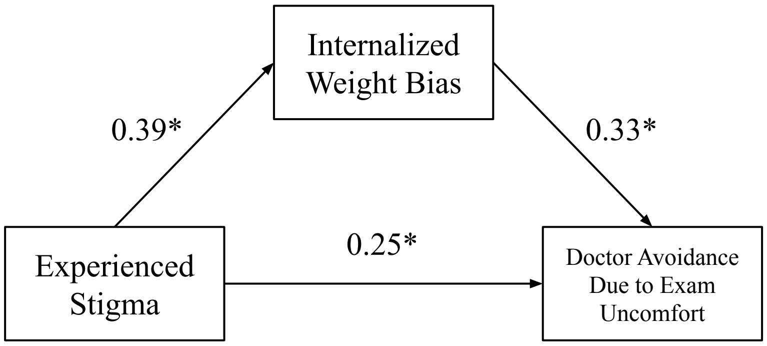

Indirect Effect = 0.13, 99% CI: 0.09 to 0.17

Figure 3b. Indirect effect of experienced stigma, **Canada**

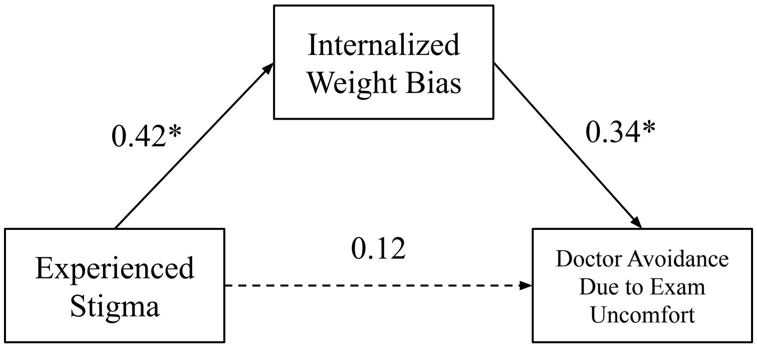

Indirect Effect = 0.14, 99% CI: 0.10 to 0.18

Figure 3c. Indirect effect of experienced stigma, **France**

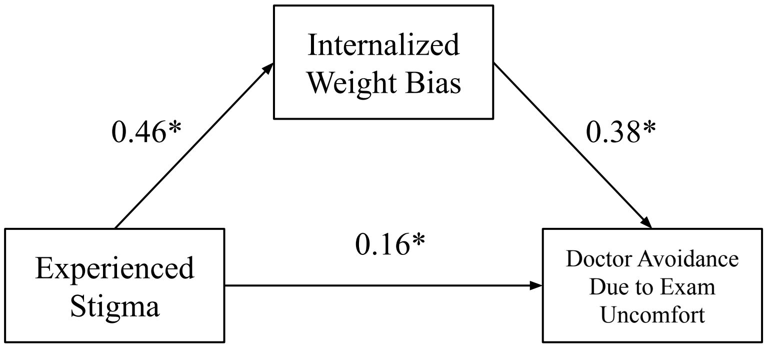

Indirect Effect = 0.18, 99% CI: 0.13 to 0.22

Figure 3d. Indirect effect of experienced stigma, **Germany**

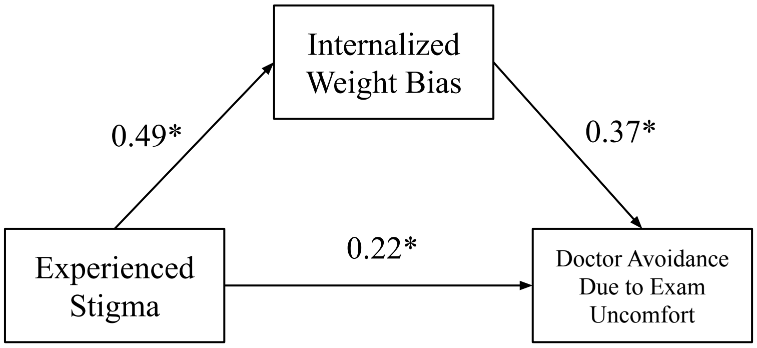

Indirect Effect = 0.18, 99% CI: 0.14 to 0.23

Figure 3e. Indirect effect of experienced stigma, **United Kingdom**

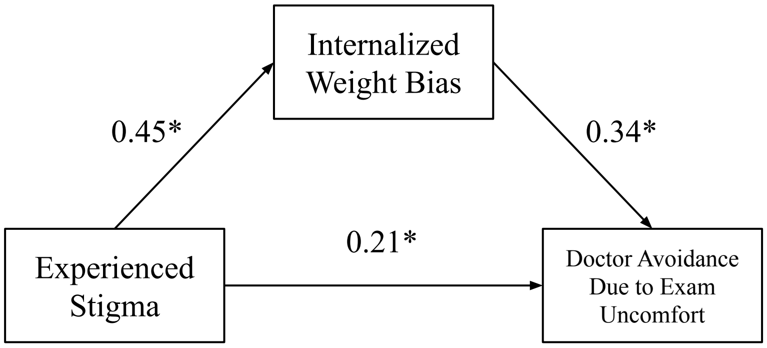

Indirect Effect = 0.15, 99% CI: 0.11 to 0.19

Figure 3f. Indirect effect of experienced stigma, **United States**
