# Supplementary material for: The roles of experienced and internalized weight stigma in healthcare experiences: Perspectives of adults engaged in weight management across six countries
Source: PLoS One. 2021 Jun 1;16(6):e0251566. doi: 10.1371/journal.pone.0251566 (PMC8168902; doi:10.1371/journal.pone.0251566)
Supplement: S3 Fig — Covariates included age, sex, educational attainment, BMI, WW membership duration, WW membership type. *p≤.001. (PDF) [file pone.0251566.s003.pdf]

Figure 4. Standardized effect estimates of experienced weight stigma on doctor listening carefully to patient in last 12 months through internalized weight bias, separately for each country. Covariates included age, sex, educational attainment, BMI, WW membership duration, WW membership type. **p* ≤ .001.

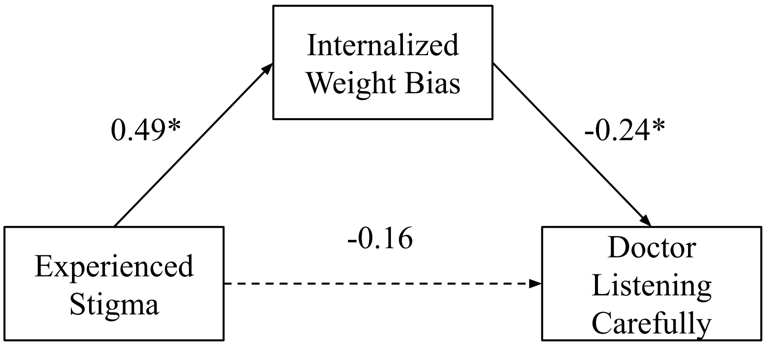

Indirect Effect = -0.12, 99% CI: -0.18 to -0.06

Figure 4a. Indirect effect of experienced stigma, **Australia**

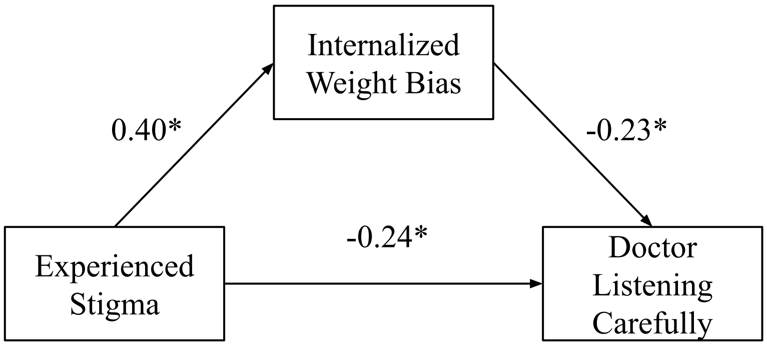

Indirect Effect = -0.09, 99% CI: -0.13 to -0.05

Figure 4b. Indirect effect of experienced stigma, **Canada**

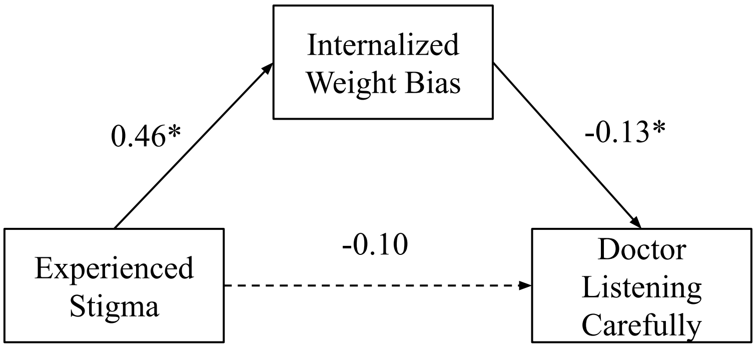

Indirect Effect = -0.06, 99% CI: -0.10 to -0.02

Figure 4c. Indirect effect of experienced stigma, **France**

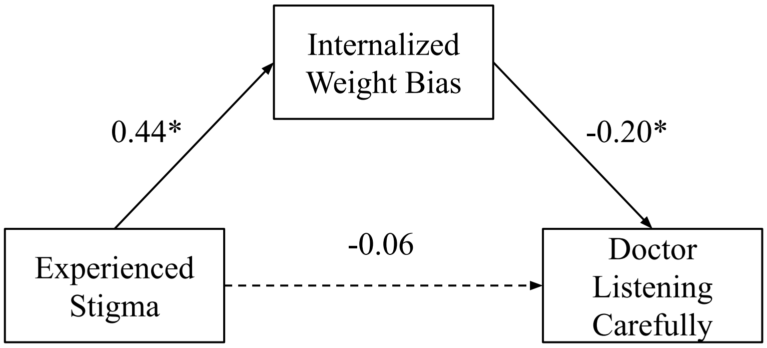

Indirect Effect = -0.09, 99% CI: -0.12 to -0.05

Figure 4d. Indirect effect of experienced stigma, **Germany**

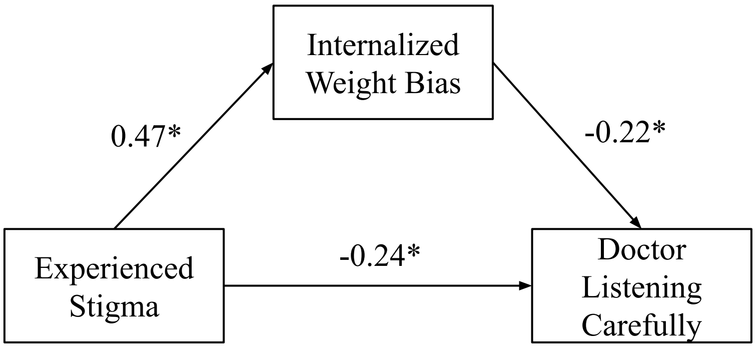

Indirect Effect = -0.10, 99% CI: -0.15 to -0.06

Figure 4e. Indirect effect of experienced stigma, **United Kingdom**

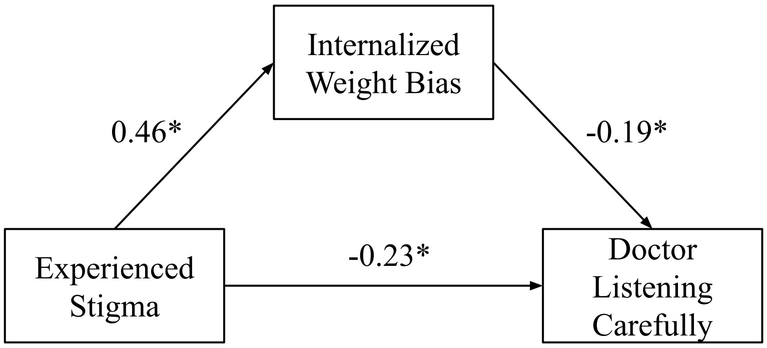

Indirect Effect = -0.09, 99% CI: -0.13 to -0.05

Figure 4f. Indirect effect of experienced stigma, **United States**

Note. Estimated models include only individuals who indicated needing medical care in the last year. (A similar pattern of results emerged when examining the associations among the full sample).
