# Supplementary material for: The roles of experienced and internalized weight stigma in healthcare experiences: Perspectives of adults engaged in weight management across six countries
Source: PLoS One. 2021 Jun 1;16(6):e0251566. doi: 10.1371/journal.pone.0251566 (PMC8168902; doi:10.1371/journal.pone.0251566)
Supplement: S7 Fig — Covariates included age, sex, educational attainment, BMI, WW membership duration, WW membership type. *p≤.001. (PDF) [file pone.0251566.s007.pdf]

Figure 8. Standardized effect estimates of experienced weight stigma on general avoidance of doctor through internalized weight bias, separately for each country. Covariates included age, sex, educational attainment, BMI, WW membership duration, WW membership type. *$p \leq .001$.

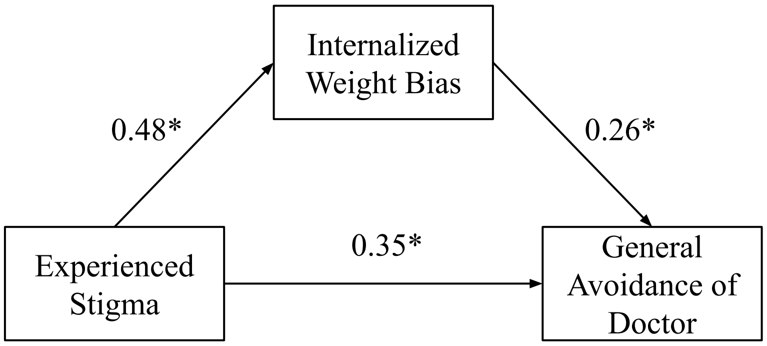

Indirect Effect = 0.13, 99% CI: 0.07 to 0.19

Figure 8a. Indirect effect of experienced stigma, **Australia**

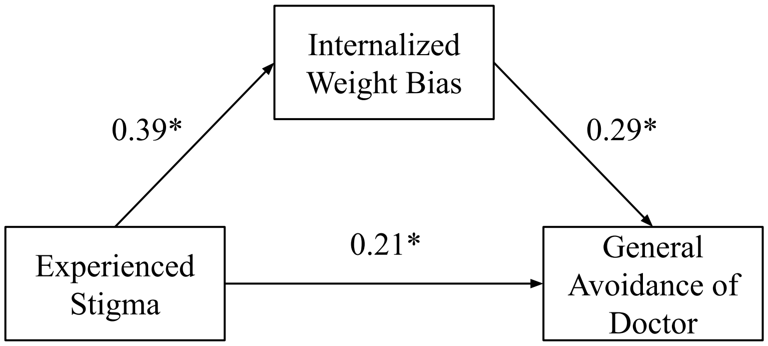

Indirect Effect = 0.12, 99% CI: 0.08 to 0.15

Figure 8b. Indirect effect of experienced stigma, **Canada**

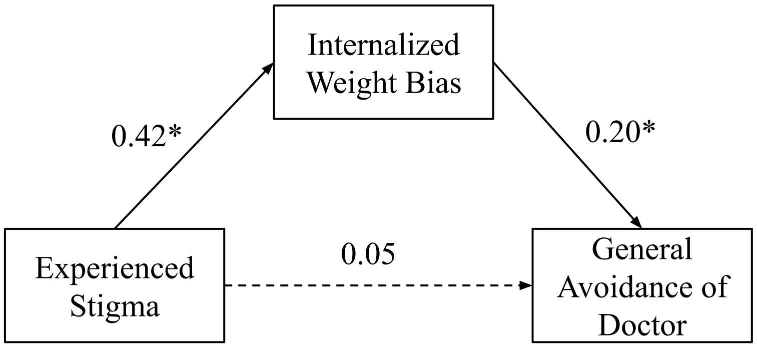

Indirect Effect = 0.09, 99% CI: 0.05 to 0.12

Figure 8c. Indirect effect of experienced stigma, **France**

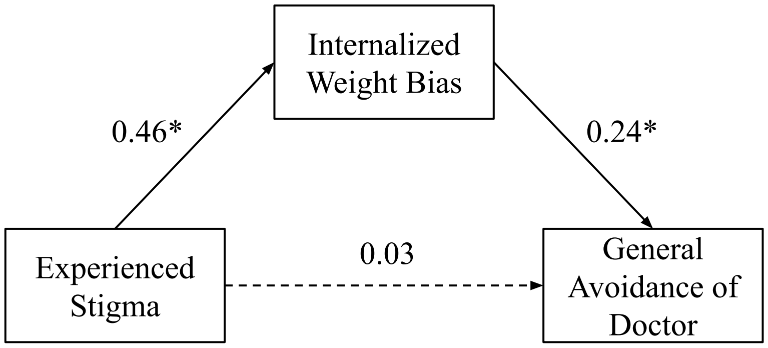

Indirect Effect = 0.11, 99% CI: 0.07 to 0.15

Figure 8d. Indirect effect of experienced stigma, **Germany**

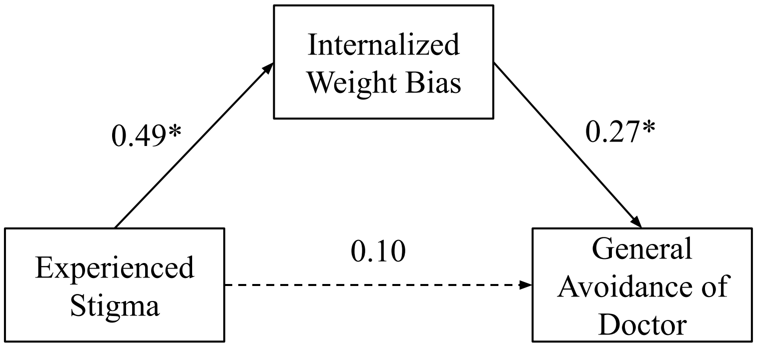

Indirect Effect = 0.13, 99% CI: 0.08 to 0.18

Figure 8e. Indirect effect of experienced stigma, **United Kingdom**

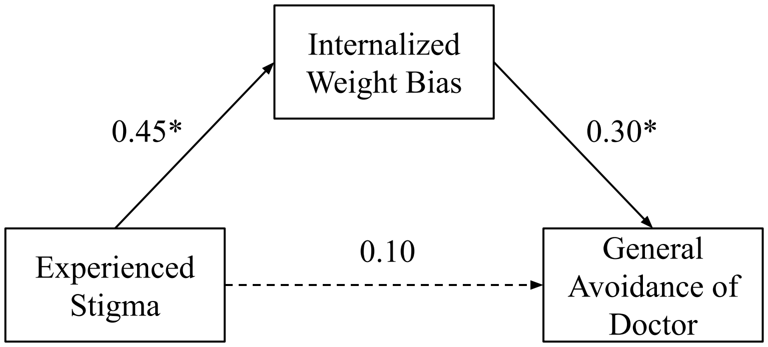

Indirect Effect = 0.13, 99% CI: 0.09 to 0.18

Figure 8f. Indirect effect of experienced stigma, **United States**
